# A deep 96-well plate RBC storage platform for high-throughput screening of novel storage solutions

**DOI:** 10.3389/fphys.2022.1004936

**Published:** 2022-10-04

**Authors:** Maria Nikulina, Travis Nemkov, Angelo D'Alessandro, Peter Gaccione, Tatsuro Yoshida

**Affiliations:** ^1^ Hemanext, Lexington, MA, United States; ^2^ Omix Technologies, Aurora, CO, United States; ^3^ University of Colorado Anschutz Medical Campus, Aurora, CO, United States; ^4^ Peter Gaccione Associates, Dennis, MA, United States

**Keywords:** blood component preparation, additive solution, red blood cell, high throughput methods, screening study

## Abstract

**Background:** Red blood cell (RBC) storage solutions, also known as additive solutions (ASs), first developed in the 1970s, enable extended storage of RBCs. Unfortunately, the advancements in this field have been limited, due to labor intensive and time-consuming serial *in vitro* and *in vivo* testing, coupled with very high commercialization hurdles. This study examines the utility of deep 96-well plates for preliminary screenings of novel ASs through comparison of RBC storage with the standard PVC bags in terms of hemolysis and ATP levels, under both normoxic (N) and hypoxic/hypocapnic (H) storage conditions. The necessity for the presence of DEHP, normally provided by PVC bags, is also examined.

**Materials and methods:** A pool of 2 ABO compatible RBC units was split between a bag and a plate. Each plate well contained either 1, 2 or 0 PVC strips cut from standard storage bags to supply DEHP. The H bags and plates were processed in an anaerobic glovebox and stored in O_2_ barrier bags. Hemolysis and ATP were measured bi-weekly using standard methods.

**Results:** Final ATP and hemolysis values for the plate-stored RBCs were comparable to the typical values observed for 6-week storage of leukoreduced AS-3 RBCs in PVC bags under both N and H conditions. Hemolysis was below FDA and EU benchmarks of 1% and 0.8%, respectively, and excluding DEHP from plates during storage, resulted in an inconsequential increase when compared to bag samples.

**Discussion:** In combination with high-throughput metabolomics workflow, this platform provides a highly efficient preliminary screening platform to accelerate the initial testing and consequent development of novel RBC ASs.

## Introduction

Additive solutions (ASs) were developed to replace plasma in component-separated red blood cells (RBCs). The first of these solutions, developed and documented in 1978, was saline-adenine-glucose (SAG). Over time, ASs evolved to increase length of storage, maintain the required metabolic activity, and minimize storage lesions for RBCs (Hess and Greenwalt, 2002; Hess, 2006; Sparrow, 2012). However, the overwhelming majority of licensed and commercialized ASs have been different variations of the original SAG solution created by adding combination of mannitol, phosphate, citrate, and guanosine or adjusting osmolarity and pH (Sparrow, 2012; D'Amici et al., 2012). No new ASs have been adopted by blood banks since AS-3 and PAGGSM in the early 2000s (Sparrow, 2012; de Korte, 2016). There are several causes for this lack of progress in the field; i) high regulatory clearance hurdles for developing solutions that are infused intravenously to millions of patients annually; ii) a lack of clinical data demonstrating compelling benefits for adoption of the newly proposed additives; and iii) the typical method of development of RBC ASs which is limited to modifying existing solutions in serial fashion showing incremental biochemical benefits, which are not sufficient commercially to justify proceeding to an expensive and time-consuming clinical outcome study (Greenwalt et al., 1984; Hess and Greenwalt, 2002; Sparrow, 2012). The serial approach is also highly time consuming and labor-intensive and thus has precluded development of radically improved additive solutions that would in turn commercially justify the expensive and prolonged efforts necessary to obtain regulatory clearance followed by clinical trials, then validation and adoption at blood establishments (Sparrow, 2012).

Recently, mass spectrometry-based omics technologies have provided a quantum leap in the capability to comprehensively analyze storage lesion development and consequently enlarge the scope of potential AS formulations (D'Alessandro et al., 2015a; D'Alessandro et al., 2015b; Rolfsson et al., 2017). In order to address the issues listed above, take advantage of the recent developments and accelerate the pace of investigations, we developed a high-throughput RBC storage and analysis platform that permits for the preliminary screening of a large number of RBC samples stored in a variety of AS formulations simultaneously.

To improve the efficiency of the initial screening stage, we examined the use of deep 96-well plates for RBC storage in various ASs using hemolysis and ATP as the primary evaluation metrics. These parameters were chosen as they are easily adopted to a 96-well workflow and allow for a sufficiently comprehensive initial characterization of the novel ASs: ingredients incompatible with RBC storage are screened out by hemolysis, and gross metabolic effects are identified by ATP levels.

One of the known consequences of storing RBCs in conventional polyvinyl chloride (PVC) storage bags is that the diethylhexyl phthalate (DEHP) plasticizer migrates from the PVC into the RBC membranes (Serrano et al., 2015), stabilizing them and in turn reducing hemolysis during storage (Hill et al., 2001; Hess, 2006; Serrano et al., 2015). Knowing that the polypropylene deep-well storage plates contain no plasticizers, this study also focused on investigating the effects of including PVC strips cut from standard storage bags to supply DEHP in the plate wells. Specifically, we compared RBC storage in small standard PVC bags and in the commonly available 2-ml polypropylene 96-well storage plates. The goal of this study was to demonstrate a sufficient minimal equivalence of the 96-well plate storage to small PVC bag storage for RBCs, which would in turn allow for its use in preliminary screenings of ASs. This study was conducted under both the conventional normoxic (N) condition and hypoxic/hypocapnic (H) storage condition. The study was designed to investigate RBC samples stored under the H condition as well as the standard N storage condition because; i) H storage has been shown to significantly enhance the quality of stored RBCs (Han et al., 2010; Dumont et al., 2016; Nemkov et al., 2022); and ii) the currently approved ASs are not optimized for H storage.

The full utility of this approach is realized when the stored RBCs plates are subsequently processed using the high-throughput metabolomics/lipidomics workflow. Nemkov and colleagues recently demonstrated the feasibility of this approach by analyzing RBC plate samples generated by this platform to examine the overall metabolic and oxidative status of stored RBCs in AS-3 spiked with five supplemented compounds (Nemkov et al., 2022).

## Materials and methods

### Red blood cell storage

**FIGURE 1:**
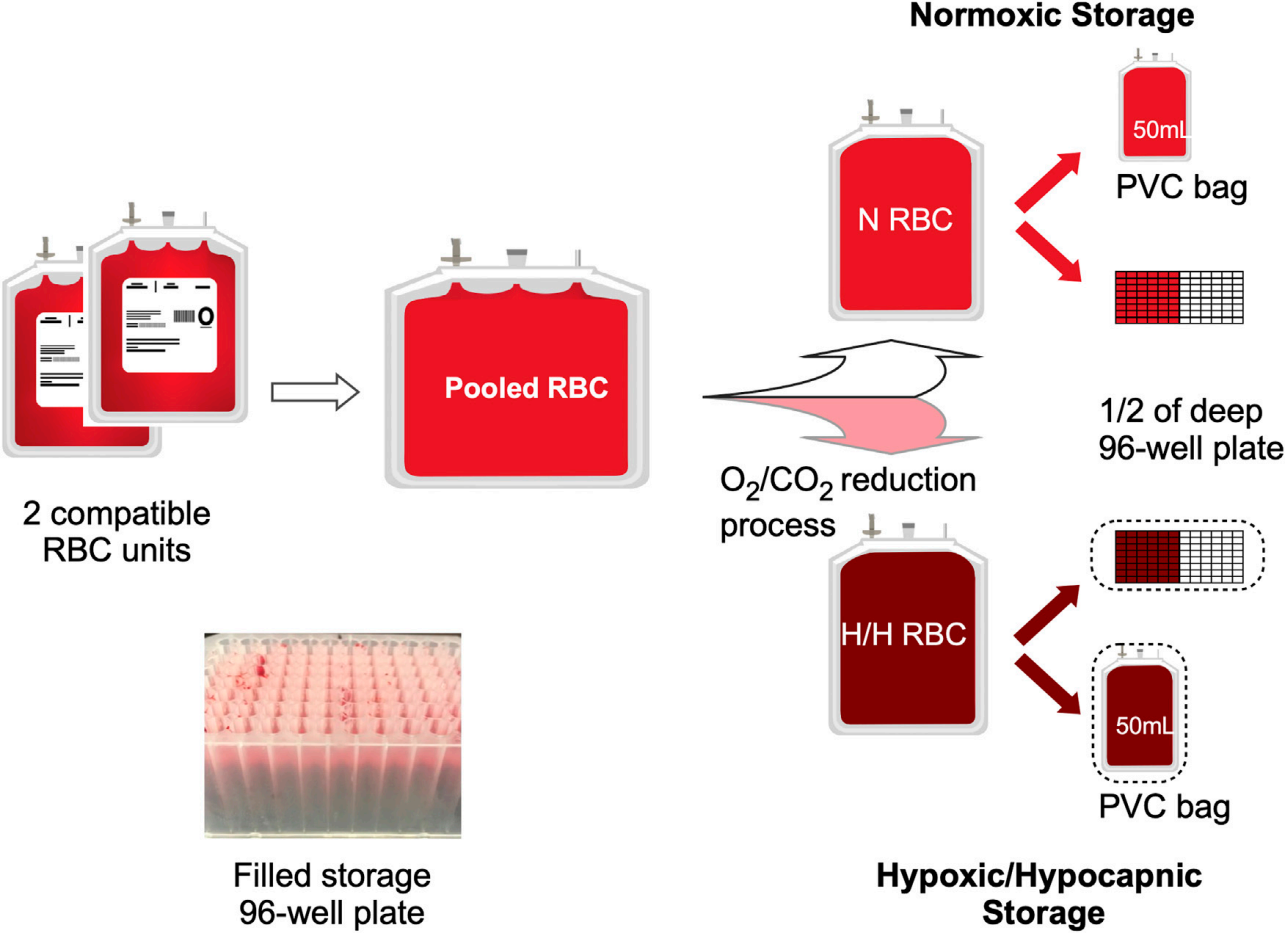
Experimental set-up. The diagram shows the processing and splitting procedure for one pool of 2 compatible RBC units. One half of the split pool remained N, and the second half was processed for O_2_/CO_2_ reduction (H). Each N or H pool was then plated into a half of the 96-well 2 ml storage plate as well as a PVC bag. (Insert) Image of set-up sample plate.

RBC or whole blood (WB) units were procured within 24 h of collection from Rhode Island Blood Center (RIBC, Providence RI) over three separate days (Group A, B and C). Due to unavailability of standard leukoreduced RBCs (LR-RBC) in AS-3 at RIBC, for Group A we received 4 pairs of compatible LR-RBC units in AS-3 prepared from CPD whole blood at RIBC and delivered overnight. For both Group B and C, we received 4 pairs of compatible units of WB in CPD from RIBC collected on the same day that were then processed in-house to produce 4 pools of LR RBCs in AS-3. The WB in CPD (BioFlex, Fenwal) was leukoreduced by a sterilely attached WB LR filter and then centrifuged (Sorvall RC 3BP + Centrifuge, Thermo Scientific) for 7 min at 3600 G. AS-3 was then added to the packed RBCs. Both the LR filter and the AS-3 used were from the Haemonetics kit (Haemonetics 126-62). Each LR RBC unit was tested for sickle cell traits with the SickleDex testing kit (Streck, Omaha NE) before pooling. The units in group A-C were pooled and split as shown in Figure 1. For each group the process was repeated for 4 RBC pools to obtain 12 pools. Normoxic units were processed in a laminar flow hood (Thermo Scientific) and hypoxic units were processed in a N_2_ (Industrial Grade, Airgas) filled glovebox (Terra Universal, CA) maintained at < 1% pO_2_. After 2 ml of RBC suspension was dispensed into each of the wells in the deep polypropylene 96-well storage plates (Greiner Bio-One PP-Masterblock 96 Well. 780286-FD, Monroe NC), the N and H plates were sealed using metallic seals (SILVERseal, Greiner Bio-One GmbH, Monroe NC). H bags and plates were stored in oxygen barrier bags (O_2_ Barrier film Z, Rollprint, Addison IL) with O_2_ sorbent (SS200, Mitsubishi Gas Chemical, NY) and sealed using a bar heat sealer. All bags and plates were stored under 4°C in a blood storage refrigerator (ThermoFisher).

PVC strips cut from standard PVC blood storage bags (∼1 cm^2^ (∼6 mm × 9 mm) from Fenwal Transfer Pack 4R2032. Fresenius-Kabi, Lake Zurich IL) were included in the wells of the storage plates to introduce DEHP. PVC strips (0, 1 or 2) were dropped into their respective wells in the polypropylene 2 ml storage plates and then autoclaved. Including 1 strip per 2 ml RBC in a well simulated ∼71% (and ∼142% for two strips) of PVC exposure estimated for the standard RBCs stored in a PVC storage bag (see Supplement for calculations). For bag storage under H conditions, O_2_ content of the small PVC storage bags (150 ml Transfer Pack, Fresenius Kabi REF:4R2001, Lake Zurich IL) was depleted by placing them under anaerobic conditions for several days to prevent re-oxygenation of the hypoxic blood upon filling.

### Red blood cell sampling

RBCs were sampled on day 0, 14, 28 and 42. N samples were sampled in the laminar flow hood. H samples were sampled in the N_2_-filled glovebox. Bags were sampled using syringes with septum ports, while plates were sampled using multichannel pipettes (Eppendorf Enfield, CT) by transferring the designated volumes into the respective plates (see Supplement for detailed procedures). sO_2_, pCO_2_, pH, lactate, K^+^, Na^+^ were measured at 37°C by the ABL90 Flex Plus co-oximeter (Radiometer, Brea CA). ATP for samples in Pool A and B was measured bi-weekly with the Hexokinase Kit (DiaSys Diagnostic Systems GmbH, Germany) adopted for 96-well workflow (see Supplement for details). Hemolysis for all samples was estimated from supernatant hemoglobin (sHb) measurement bi-weekly based on the Harboe direct spectrophotometric method with Allen correction (Han et al., 2010) adopted for 96-well plate workflow. The spectroscopic assays were carried out using the BioTek Epoch 2 reader with Gen TS Microplate Data Collection and Analysis Software (BioTek Instruments, Inc., Vermont). Hemolysis was estimated (e-hemolysis) from sHb using a correlation function:

e−hemolysis (%)=sHb (mg/dL) ∗ 0.002

Derived from an existing data set of sHb vs. calculated hemolysis using the spun hematocrit method (see Supplement for details). High throughput metabolomics plates (Greiner Bio-One 96 well PP microplate, 651,204, Monroe NC) were also prepared for a separate study; 0.05 ml of RBCs was transferred and frozen at −80°C. For H samples, after the RBCs were transferred, the plates were covered with metallic seals placed in barrier bags with oxygen sorbent, frozen on dry ice in a N_2_-filled glove box and stored at −80°C.

### Statistical analysis

Both hemolysis and ATP values were analyzed separately using a mixed model analysis of variance, with a random effect of RBC pool, to estimate the effects of storage method and differences over time. In all analyses, the dependent variable was hemolysis or ATP at weeks 2, 4, and 6, and the independent fixed effect variables were baseline pool value, time point (week 2, 4, 6), process method (N or H), storage method (0, 1, or 2 PVC strips, or bag), interaction of time point and storage method, interaction of process method and storage method, interaction of process method and time point, and interaction of process method and storage method and time point. In either case Bonferroni multiple adjustment procedures were implemented.

## Results

### Red blood cell storage under N and H conditions: sO_2_ and pCO_2_

**FIGURE 2:**
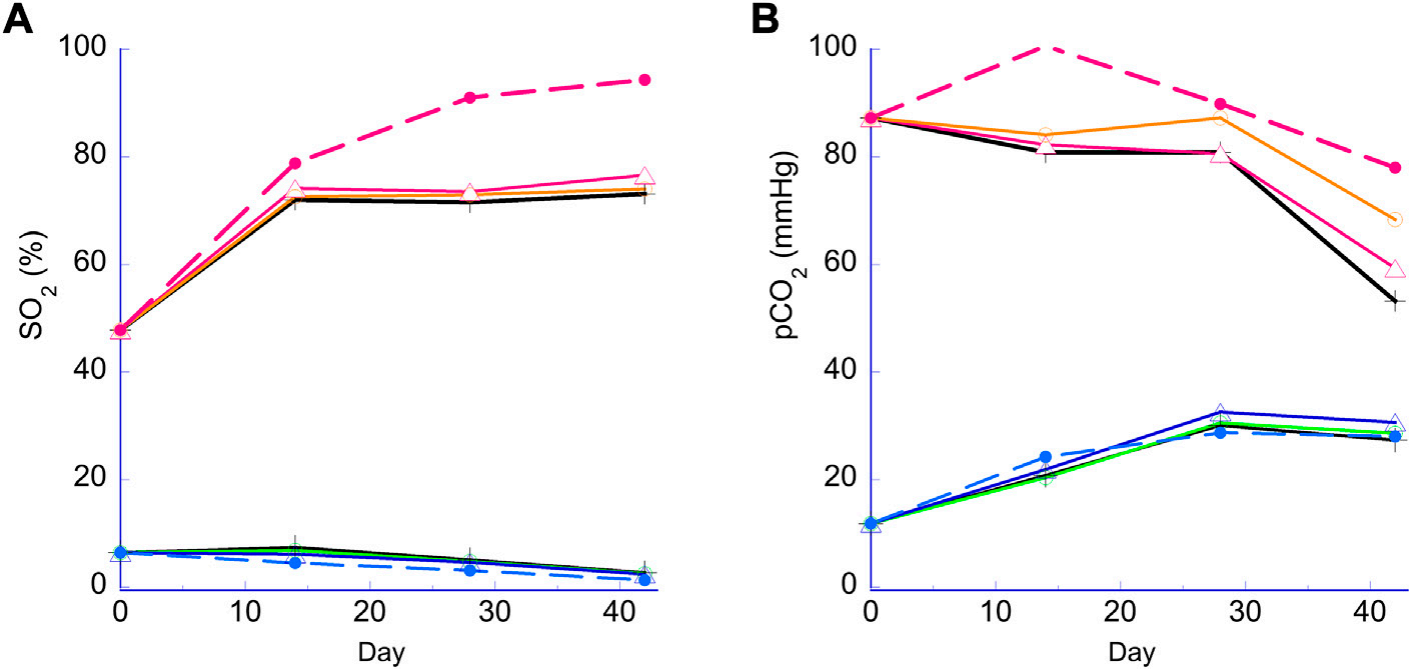
Hemoglobin oxygen saturation (sO_2_) and pCO_2_ during storage. Panel **(A)** sO_2_ (%). Panel **(B)** pCO_2_ (mmHg). Lower lines (<20% sO_2_ or 40mmHg pCO_2_), H storage; higher lines (>40% or >40mmHg), N storage. Broken lines, PVC bag; open triangle, 2 PVC strips; open circle, 1 PVC strip; cross, no PVC strip.

Figure 2 illustrates the hemoglobin oxygen saturation (sO_2_) and pCO_2_ values for N and H samples. For N samples, an increased sO_2_ was observed due to the diffusion of O_2_ from ambient air into the RBC suspension during storage, especially notable for the bag. The sO_2_ gain was limited for the RBCs in the 96-well plates sealed at the top with a metallic seal. The large increase at the 2-week measurement was mainly due to initial oxygenation during the transfer of the RBCs into the bag and wells. This effect was largely absent from H RBCs as H RBCs were transfered in an anaerobic chamber. Anaerobically stored H samples with oxygen sorbent showed decreasing sO_2_ over the course of storage from the gradual loss of O_2_ through the container (∼6%–∼2% over 6 weeks, more pronounced with bag; Figure 2A). For pCO_2_, samples stored under the N condition showed a decreasing trend as CO_2_ escaped into ambient air and the H stored samples showed an increasing trend as CO_2_ was produced by the pentose pathway from the initial depleted state. Both these trends for sO_2_ and pCO_2_ were similar to previous observations (Dumont et al., 2016) and are a result of the two different storage conditions. Within the N plate samples, sO_2_ and pCO_2_ values were affected to a small extent by storage method (bag or PVC strips) at week 4 and 6.

### Hemolysis

The estimated hemolysis for all N and H samples was well below the 1% FDA mark and 0.8% EU requirement, the highest being about half of the FDA benchmark at 0.5% for both H and N storage. All individual e-hemolysis profiles for samples stored in the 96-well plates showed a similar trend. A minor difference was observed for the H PVC bag samples with a recorded slower increase in e-hemolysis (Table 1A). Including 1 strip per 2 ml RBCs in a well achieved ∼71% (and 142% for two strips) of estimated PVC exposure for the standard RBC stored in a PVC storage bag (AS-3 RBC Storage bag Haemonetics, Braintree, MA).

**TABLE 1:** Descriptic statistics for ATP and e-hemolysis.

1A: e-Hemolysis (%)									
Measure	Week	H (Hypoxic)	H	H	H	N (Normoxic)	N	N	N
		PVC 0	PVC 1	PVC 2	Bag	PVC 0	PVC 1	PVC 2	Bag
n		12	12	12	10	12	12	12	12
Mean (Std)	0	0.16 (0.113)	0.16 (0.113)	0.16 (0.113)	0.14 (0.115)	0.10 (0.085)	0.10 (0.085)	0.10 (0.085)	0.10 (0.085)
Median		0.11	0.11	0.11	0.1	0.07	0.07	0.07	0.07
Min,Max		0.05,0.43	0.05,0.43	0.05,0.43	0.05,0.43	0.03,0.32	0.03,0.32	0.03,0.32	0.03,0.32
n		12	12	12	12	12	11	12	12
Mean (Std)	2	0.22 (0.124)	0.25 (0.183)	0.26 (0.152)	0.21 (0.113)	0.21 (0.124)	0.20 (0.139)	0.19 (0.124)	0.22 (0.106)
Median		0.17	0.17	0.21	0.18	0.17	0.14	0.19	0.22
Min,Max		0.11,0.55	0.10,0.66	0.09,0.58	0.09,0.49	0.07,0.44	0.05,0.48	0.07,0.50	0.07,0.41
n		12	12	12	12	12	12	12	11
Mean (Std)	4	0.35 (0.136)	0.34 (0.188)	0.33 (0.123)	0.23 (0.085)	0.28 (0.106)	0.26 (0.208)	0.28 (0.140)	0.30 (0.123)
Median		0.35	0.31	0.32	0.23	0.28	0.18	0.24	0.25
Min,Max		0.15,0.64	0.16,0.88	0.14,0.60	0.12,0.47	0.12,0.47	0.07,0.70	0.09,0.62	0.15,0.54
n		12	12	11	12	12	12	12	12
Mean (Std)	6	0.51 (0.164)	0.41 (0.164)	0.46 (0.190)	0.30 (0.151)	0.50 (0.202)	0.39 (0.204)	0.38 (0.174)	0.40 (0.132)
Median		0.47	0.35	0.45	0.29	0.42	0.35	0.35	0.37
Min,Max		0.34,0.89	0.19,0.73	0.20,0.82	0.12,0.59	0.28,0.98	0.16,0.80	0.15,0.74	0.24,0.72

B:ATP (μmol/gHb)									
Measure	Week	H (Hypoxic)	H	H	H	N (Normoxic)	N	N	N
		PVC 0	PVC 1	PVC 2	Bag	PVC 0	PVC 1	PVC 2	Bag
n		8	8	8	8	8	8	8	8
Mean (Std)	0	5.70 (0.551)	5.83 (0.579)	5.95 (0.785)	6.08 (1.071)	5.02 (1.523)	5.02 (1.523)	5.02 (1.523)	5.02 (1.523)
Median		5.67	5.96	5.96	5.96	4.96	4.96	4.96	4.96
Min,Max		4.85,6.48	4.85,6.48	4.85,7.37	4.85,8.37	3.53,6.68	3.53,6.68	3.53,6.68	3.53,6.68
n		8	8	8	8	8	8	8	8
Mean (Std)	2	5.63 (0.658)	5.98 (0.660)	6.07 (0.510)	5.70 (1.729)	5.18 (0.467)	5.57 (0.456)	5.78 (0.517)	5.87 (0.841)
Median		5.6	5.88	6.06	6.04	5.31	5.68	6	5.9
Min,Max		4.64,6.59	5.19,7.05	5.33,6.73	1.56,6.94	4.32,5.70	4.73,6.17	4.79,6.23	4.71,7.15
n		8	8	8	8	8	8	8	8
Mean (Std)	6	5.05 (0.256)	5.38 (0.414)	5.58 (0.610)	5.79 (0.646)	4.44 (0.617)	4.70 (0.594)	4.81 (0.624)	5.37 (0.678)
Median		4.9	5.43	5.7	5.93	4.71	5.04	4.92	5.14
Min,Max		4.84,5.44	4.63,5.85	4.23,6.20	4.71,6.63	3.08,5.03	3.87,5.28	3.94,5.72	4.45,6.33
n		8	8	8	8	8	8	8	8
Mean (Std)	6	3.33 (0.466)	3.49 (0.599)	3.71 (0.495)	3.69 (0.548)	2.82 (0.277)	3.07 (0.287)	3.26 (0.489)	3.17 (0.418)
Median		3.38	3.56	3.74	3.81	2.89	3.04	3.37	3.15
Min,Max		2.37,3.96	2.61,4.65	2.89,4.58	2.99,4.57	2.27,3.12	2.71,3.58	2.42,4.03	2.50,3.82

A mixed model of variance was constructed (Table 2) in order to further examine the effects of plate storage and DEHP supplied by 1 or 2 PVC strips on the stored RBCs. No significant difference was observed between N and H storage samples overall (time and PVC/bag) (Table 2A). For H storage, the analysis of overall e-hemolysis for the bag estimated a small but significant reduction, with the largest estimated difference of 0.11% between the bag and the 0 PVC samples (*p* ≤ 0.0001). As shown in Table 2C, at week 6, significant differences were estimated between the bag (0.28%), 0 PVC strip (0.49%, (*p* ≤ 0.0001)), as well as between the bag and 2 PVC strips (0.44%, *p* < 0.023). The estimated differences were not significant among the H samples in 96-well plate regardless of the PVC strip count (Table 2C). For N storage, no significant overall differences for hemolysis were estimated, even at the end of storage, for the bag sample and 96-well plate samples with 0, 1 and 2 PVC strips (Table 3D).

**TABLE 2:** Hemolysis: Mean estimated values from the model.

A. Overall estimated differences for storage condition (H vs. N)			
Independent variables			Difference of estimated means (*p* values shown only for *p* < 0.05)
Overall wk (2,4,6) and PVC (0,1,2,bag)			H vs. N
H	overall wk	0.304	−0.01
N		0.318	

B. Weekly estimated differences for PVC levels (H vs. N)									
Independent variables						Difference of estimated means (*p* values shown only for p < 0.05)			
Storage	Week	Estimated means; PVC strip or bag				H vs. N			
		PVC 0	PVC 1	PVC 2	Bag	PVC 0	PVC 1	PVC 2	Bag
H	Overall Wk	0.340	0.316	0.327	0.233	−0.01	0.01	0.03	−0.09
N		0.348	0.301	0.301	0.320				

C. Weekly estimated differences for PVC levels under hypoxic (H) storage											
Independent variables						Difference of estimated means (*p* values shown only for p < 0.05)					
Storage	Week	Estimated means; PVC strip or bag				PVC strip or bag					
		PVC 0	PVC 1	PVC 2	Bag	PVC 0_vs._1	PVC 0_vs._2	PVC 0_vs._Bag	PVC 1_vs._2	PVC 1_vs._Bag	PVC 2_vs._Bag
H	overall wk	0.340	0.316	0.327	0.233	0.02	0.01	0.11 (ps0.0001)	−0.01	0.08 (ps0.0013)	0.09 (ps0.001)
H	2	0.200	0.232	0.235	0.198	−0.03	−0.03	0	0	0.03	0.04
H	4	0.333	0.324	0.306	0.218	0.01	0.03	0.11	0.02	0.11	0.09
H	6	0.486	0.392	0.441	0.281	0.09	0.05	0.21 (ps0.0001)	−0.05	0.11	0.16 (ps0.023)

D. Weekly estimated differences for PVC levels under normoxic (N) storage											
Independent variables						Difference of estimated means (*p* values shown only for p < 0.05)					
Storage	Week	Estimated means. PVC ship o bag				PVC ship or bag					
		PVC 0	PVC 1	PVC 2	Bag	PVC 0_vs._1	PVC 0_vs._2	PVC 0_vs._Bag	PVC 1_vs._2	PVC 1_vs._Bag	PVC 2_vs._Bag
N	overall wk	0.348	0.301	0.301	0.320	0.05	0.05	0.03	0	−0.02	−0.02
N	2	0.230	0.211	0.213	0.235	0.02	0.02	0	0	−0.02	−0.02
N	4	0.298	0.280	0.296	0.309	0.02	0	−0.01	−0.02	−0.03	−0.01
N	6	0.517	0.412	0.396	0.417	0.1	0.12	0.1	0.02	0	−0.02

**TABLE 3:** ATP: Mean estimated values from the model.

A. Overall estimated differences for storage condition (H vs. N)			
Independent variables			Difference of estimated means (*p* values shown only for *p* < 0.05)
Overall wk (2,4,6) and PVC (0,1,2.bag)			H vs. N
H	overall wk	4.97	0.48 (p50.0001)
N		4.49	

B. Weekly estimated differences for PVC levels (H vs. N									
Independent variables						Difference of estimated means (*p* values shown only for p < 0.05)			
Storage	Week	Estimated means: PVC strip or bag				H vs. N			
		PVC 0	PVC 1	PVC 2	Bag	PVC 0	PVC 1	PVC 2	Bag
H	overall wk	4.68	4.97	5.13	5.08	0.56 (*p* < 0.05)	0.53	0.54	0.29
N		4.13	4.43	4.60	4.79				

C. Weekly estimated differences for PVC levels under hypoxic (H) storage											
Independent variables						Difference of estimated moans (*p* values shown only for p < 0.05)					
Storage	Week	Estimated means: PVC strip or bag				PVC strip or bag					
		PVC 0	PVC 1	PVC 2	Bag	PVC 0_vs._1	PVC 0_vs._2	PVC 0_vs._Bag	PVC 1_vs._2	PVC 1_vs._Bag	PVC 2_vs._Bag
H	overall wk	4.68	4.97	5.13	5.08	−0.28	−0.45	−0.4	−0.17	−0.11	0.06
H	2	5.64	6.00	6.08	5.72	−0.35	−0.44	−0.08	−0.09	0.28	0.36
H	4	5.06	5.40	5.59	5.81	−0.34	−0.53	−0.74	−0.19	−0.41	−0.21
H	6	3.34	3.50	3.72	3.71	−0.16	−0.38	−0.36	−0.22	−0.21	0.02

D. Weekly estimated differences for PVC levels under normoxic (N) storage											
Independent variables						Difference of estimated moans (*p* values shown only for p < 0.05)					
Storage	Week	Estimated means: PVC strip or bag				PVC strip or bag					
		PVC 0	PVC 1	PVC 2	Bag	PVC 0_vs._1	PVC 0_vs._2	PVC 0_vs._Bag	PVC 1_vs._2	PVC 1_vs._Bag	PVC 2_vs._Bag
N	overall wk	4.13	4.43	4.60	4.79	−0.31	−0.47	−0.66 (*p* ≤0.004)	−0.16	−0.35	−0.19
N	2	5.16	5.56	5.76	5.85	−0.4	−0.6	−0.69	−0.2	−0.3	−0.09
N	4	4.42	4.69	4.79	5.35	−0.27	−0.37	−0.93	−0.11	−0.66	−0.56
N	6	2.80	3.06	3.24	3.15	−0.26	−0.44	−0.35	−0.18	−0.1	0.09

### ATP

Table 1A shows the ATP values for the N and for H RBCs throughout the 6-week storage accompanied by descriptive statistics and the results of analysis by a mixed model analysis of variance (Table 3). Higher ATP levels were observed for samples stored hypoxically, as reported previously (Yoshida and Shevkoplyas, 2010; Dumont et al., 2016; Yoshida et al., 2022) with an overall estimated difference = 0.48µmol/gHb, *p* ≤ 0.0001. The three observed ATP profiles of the samples stored in the 96-well plate with PVC strips were similar to the values and trends seen in samples stored in PVC bags, both in N and H storage conditions over the 6-week study period. There was no significant estimated difference within the H samples regardless of bag or plate storage, number of PVC strips, or time point throughout the study. Within the N samples, a small but significant estimated difference (0.66µmol/gHb *p* < 0.004) in ATP was predicted by the model for the bag storage compared to the plate storage with no PVC (Table 3).

### Other parameters

**FIGURE 3:**
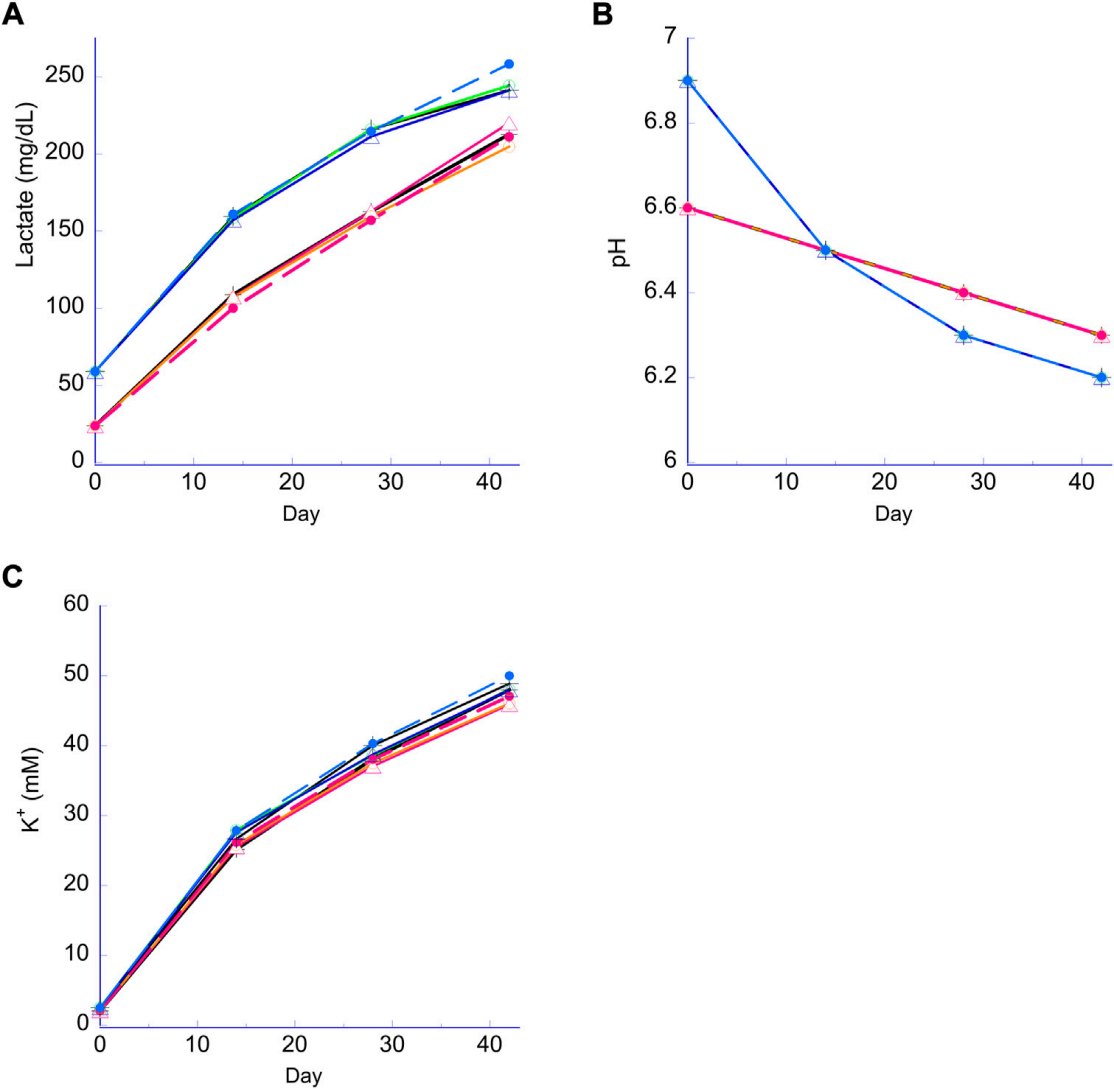
Lactate, pH and extracellular K+. Panel **(A)** lactate for both the N RBC (lower lines) and H RBC (higher lines). Panel **(B)** pH for both the N RBC (family of lines stating lower at Day 0); H RBC (family of lines starting higher then cross over at week 2); Panel **(C)** K^+^ (differences are negligible and within precision of the analyzer.) Broken lines, PVC bag; open triangle, 2 PVC strips; open circle, 1 PVC strip; cross, no PVC strips.

Lactate, pH and K^+^ all followed their respective expected trends for the N and H storage samples, and none of these parameters were significantly affected when the bag samples versus the plate samples were compared within the N or H storage conditions, regardless of the quantity of PVC strips or storage in the bag (Figure 3). Lactate levels in both the H and N samples increased over the course of the study (Figure 3A) with higher levels observed in the H storage as reported previously. The pH profiles (Figure 3B) followed the expected trend with a higher initial pH for the H condition from pCO_2_ reduction, followed by a faster drop due to a higher rate of lactate production (Dumont et al., 2016; D'Alessandro et al., 2017). The rate of cell-free K^+^ increase was indistinguishable for all samples (Figure 3C).

## Discussion

As blood transfusions are one of the most common patient procedures performed in hospitals (Pfuntner et al., 2013) and red blood cells (RBCs) are the most transfused component (World_Health_Organization, 2017), there has been renewed interest in the relationship between the quality of the stored RBC and post-transfusion outcomes (Zimrin and Hess, 2009; Tissot et al., 2017; Hunsicker et al., 2018; Sardar et al., 2018; DeSantis et al., 2019; Yoshida et al., 2019). Multiple investigations into RBC storage lesions, often correlated with storage duration, and subsequent patient outcomes have been conducted leading to concerns about the safety and efficacy of stored RBCs for a variety of patients (Koch et al., 2008; Wang et al., 2012; Lelubre and Vincent, 2013; Brunskill et al., 2015). While the current understanding of storage lesions is incomplete (Glynn, 2010; Rapido et al., 2017; Yoshida et al., 2019), additive solutions (ASs) specifically have long been investigated and developed as a means of reducing storage lesions for RBCs and increasing positive patient outcomes (Hess, 2006; Lelubre et al., 2009; Sparrow, 2012; Lagerberg et al., 2017). The first AS, SAG (saline, adenine and glucose), was developed in the late 1970s and quickly gave way to SAGM in 1981, which is still widely used today (Sparrow, 2012; D'Amici et al., 2012). The subsequent modifications of these original two ASs, include such licensed and commercially available ASs as AS-1, AS-5 and more recently AS-3 and PAGGSM (Hess, 2006; D'Amici et al., 2012; Zehnder et al., 2008). However, studies report that none of these solutions possess any significant advantages over the rest in terms of 24-h *in vivo* recovery and hemolysis (Hess, 2006). Numerous other experimental ASs have been reported in literature over the past 20 years (Sparrow, 2012; Hess and Greenwalt, 2002; Rolfsson et al., 2017; D'Alessandro et al., 2018; Burger et al., 2012; Radwanski et al., 2013; Cancelas et al., 2015), but they never achieved commercial adoption. This is due to the fact that their consequent improvements in stored RBCs were insufficient to overcome hurdles faced by the manufacturers for meeting the ever-increasing regulatory and manufacturing requirements coupled with the significant burdens for novel AS implementation at a large number of independent blood establishments worldwide. Additional obstacles in AS development have been the serial method of modification and investigation that requires intensive labor and time; and a lack of clinical data demonstrating sufficient AS improvement, which have limited the scope of investigation and slowed innovations that could potentially withstand commercialization challenges.

We developed the deep 96-well plate-based storage and sampling platform specifically for the initial screening stage in the development process of novel ASs to broaden the scope of investigations and accelerate their pace. This platform allows for the preliminary testing of a formulation of an AS in a single well instead of an individual PVC bag. This platform is advantageous due to its efficient use of resources in both storage and subsequent assay workflow. Additionally, it allows for a dramatic increase in the number of AS formulations that can be tested at any single time, allowing for investigations with a multitude of factors such as dosage and combinations of ingredients that is impractical otherwise.

We tested the plate storage under the regular normoxic (N) condition in ambient air, as well as under an oxygen and CO_2_ controlled condition—‘anaerobic’, or hypoxic/hypocapnic (H) condition, as the latter has been shown to produce a variety of benefits including a reduction in oxidative stress and the reprograming of RBC metabolism in beneficial ways (D'Alessandro et al., 2020) (Yoshida and Shevkoplyas, 2010). As shown in Figure 2, the main characteristic difference between the two conditions is determined by oxygen content of the RBCs measured as hemoglobin oxygen saturation (sO_2_), and pH due to pre-storage reduction of O_2_ and pCO_2_. A large and expected difference in the sO_2_ was maintained between N and H samples throughout the study (Figure 2A) as intended. Under N conditions a noticeable difference of sO_2_ was observed between the bag and the plate samples (Figure 2A), which can be attributed to the bag having a larger surface area exposed to ambient air and allowing for higher levels of oxygenation due to O_2_ diffusion during storage (Yoshida et al., 2022). Additionally, metallic foil seals were used for both types of plates, which eliminated O_2_ ingress from the top. For the N plates, only the surface area on the bottom and sides was exposed to ambient air, and due to the relatively low O_2_ permeability of the rigid polypropylene, average sO_2_ remained stable except for the initial sO_2_ increase which occurred during the filling of the wells in ambient air. There was also a small difference in sO_2_ within the H plate samples, especially between the 0 and 2 strip samples (Figure 2A). Further investigation is needed to explain this observation, although such a small difference in sO_2_ is physiologically insignificant. The typical pCO_2_ profile and difference in pCO_2_ values between the N and H conditions observed in bag stored samples (Dumont et al., 2016) were replicated in the plate platform (Figure 2B). Higher pCO_2_ levels observed for the normoxic bag sample were likely a result of higher flux through the oxidative pentose pathway triggered by higher oxygen levels.

During our study we had to account for the fact that during conventional RBC storage in PVC bags, DEHP leaches out from the PVC bags and reduces RBC hemolysis (Estep et al., 1984) (Serrano et al., 2015). Since the polypropylene plates used for RBC storage lacked DEHP, we examined the potential consequences by storing RBCs in wells containing either 0, 1 or 2 PVC strips cut from standard PVC storage bags, with 1 strip corresponding to the approximate area/volume ratio derived from standard RBC storage bags. This set-up allowed us to evaluate whether DEHP needs to be supplied during polypropylene plate storage for AS screenings for this platform to be a viable screening method (Estep et al., 1984) (Serrano et al., 2015).

We chose estimated hemolysis (e-hemolysis) and ATP as the two quality-metrics for initial AS screenings, since ingredients resulting in high hemolysis or low ATP during storage can be eliminated early prior to further investigations. These metrics were also chosen because their assays are readily available and adoptable to the 96-well platform workflow. At the end of 6-week storage when the well samples with and without DEHP were compared to each other, as well as to the conventional PVC bag samples, there was a slight yet significant increase in e-hemolysis for the samples stored with no PVC strips in plate wells. However, since end of storage e-hemolysis was 0.51 ± 0.16 (N) and 0.50 ± 0.20 (H) for the 0 strip samples, well below the 0.8% EU requirement and about half of the 1.0% FDA limit, it was concluded that the absence of DEHP does not prevent the successful use of this platform for initial AS screening studies. In anticipation of DEHP being phased out of use in blood storage bags in the near future (Razatos et al., 2022), we will conduct future initial screenings without introducing DEHP infused PVC strips.

There was a significant difference in ATP levels between the H and N (Table 1B and Supplementary Figure) samples stored under the respective conditions as previously observed in bag-based studies (Yoshida and Shevkoplyas, 2010; D'Alessandro et al., 2020). The presence of this significant difference demonstrated the translatability of this platform for both N and H storage conditions. Overall, based on the two metrics of e-hemolysis and ATP, it can be concluded that the novel storage platform is sufficiently equivalent to the standard bag method for RBC storage for the purpose of initial AS screening and usable under both H and N storage conditions.

The proposed platform’s equivalence is further demonstrated by additional biochemical parameters such as pH, lactate and K^+^ (Figure 3). As expected, due to H processing and subsequent storage, there were higher lactate levels observed for H samples when compared to N samples (Figure 3A), reflecting an enhanced glycolytic flux from H storage (Dumont et al., 2016; Yoshida and Shevkoplyas, 2010; D'Alessandro et al., 2020). Lower pCO_2_ and higher initial pH observed for H samples were mainly due to CO_2_ depletion during the O_2_ reduction process (Figure 3B) and the observed pH cross-over mid-storage (Figure 3B) can be ascribed to the increased rate of lactate accumulation during H storage (Dumont et al., 2016). On the other hand, there was no noticeable difference in K+ between any of the samples, regardless of time point, strip number, storage method (plate vs. bag) or storage condition (H or N).

Due to a limited availability of standard AS-3 RBCs, RBCs in AS-3 additive were prepared from WB collected in CPD instead of CP2D anticoagulant, resulting in a reduced initial glucose concentration (CPD WB 436 ± 37 mg/dl *n* = 47, measured in this study compared to CP2D WB 600 ± 43 mg/dl *n* = 42, data from previous studies). However, we believe that the main observations from this study remain valid as typically, more than 50% of the glucose remained after 6 weeks of storage and the observed ATP, lactate, pH and e-hemolysis profiles were comparable to those observed during typical AS-3 storage. In order to analyze the large number of samples generated in this format, hemolysis was estimated from measured sHb based on a correlation function sHb vs. hemolysis with spun hematocrit derived from more than 400 data points specifically for LR AS-3 RBC stored in H and N conditions over a 6-week period.

In order to minimize the effects of donor blood variability (Mykhailova et al., 2020; Hadjesfandiari et al., 2021) in this study, all of the metrics described above were measured and averaged across 24 units spread into 12 compatible pools. Since a novel AS needs to be universally compatible with donated RBCs, if one RBC unit in one of the pools caused high hemolysis, the measured hemolysis value for the pool would indicate the presence of an AS incompatible with at least one of the units in the pool, screening out the incompatible AS candidate at an early stage.

After the completion of the initial screening study stage utilizing the described deep 96-well plate storage method, promising candidate ingredients will be further tested in subsequent studies, first with small bags, followed by full unit final storage bags evaluated using hemolysis with spun hematocrit values.

Once a promising AS is identified, this platform can then also be adopted to screening the ASs’ universal compatibility against a large number of individual RBC units using RBCs in segments.

After narrowing down the candidate ASs to a more manageable number, they can be further investigated through more detailed studies including measurements of RBC deformability and P50, as well as more detailed metabolomics/lipidomics investigations using isotopes to elucidate the mechanism of action. The final candidate(s) can then proceed to a full-unit storage study in the final storage configuration, followed by a clinical study to determine post-transfusion recovery of stored autologous RBCs. Recently, an accompanying paper from Nemkov et al. has shown that the overall metabolic profile generated by this platform is comparable to that of RBCs stored in a standard storage bag, and successfully demonstrated the platform’s feasibility by applying it to examine the effect of spiking five different compounds into the AS-3 additive (Nemkov et al., 2022).

Limitations: We acknowledge that RBCs stored in a small PVC bag used as the comparator to the plate in this study are not identical when compared to RBCs stored as a full unit. Data presented in this study only demonstrates that a high throughput RBC storage method based on a deep 96-well plate is sufficiently equivalent to the conventional storage method with a reduced bag volume for use in preliminary screenings of ASs. The described method was not developed or proposed as a replacement for conventional storage, nor do we claim it is identical to the conventional method. The plate platform was developed to allow for a more efficient and less demanding initial screening process of novel ASs before they are tested further using the conventional bag method and subsequently *in vivo*. Due to unavailability of AS-3 RBCs at the blood supplier, whole blood was collected in CPD instead of CP2D anticoagulant, and then processed into LR-RBC in AS-3 reducing the initial glucose concentration by ∼27%, potentially affecting RBC metabolism during storage. RBC samples stored in the polypropylene storage plates without PVC strips in this study were not totally free of DEHP as the whole blood was initially collected and processed in DEHP-containing collection kits.

## Conclusion

Combined with high throughput metabolomics workflow, the deep 96-well plate-based blood storage and analysis platform can greatly broaden the scope and increase the pace of investigations and development of novel ASs optimized for DEHP-free RBC storage. This approach has the potential to hasten the development of a novel AS with significantly enhanced storage characteristics that can overcome high commercialization hurdles that have prevented the adoption of previous novel ASs.

## Data Availability

The raw data supporting the conclusions of this article will be made available by the authors, without undue reservation.
