# Supplementary material for: A deep 96-well plate RBC storage platform for high-throughput screening of novel storage solutions
Source: Front Physiol. 2022 Oct 4;13:1004936. doi: 10.3389/fphys.2022.1004936 (PMC9583842; doi:10.3389/fphys.2022.1004936)
Supplement: Supplementary file 1 [file DataSheet1.PDF]

Supplement 1: Estimated hemolysis and ATP

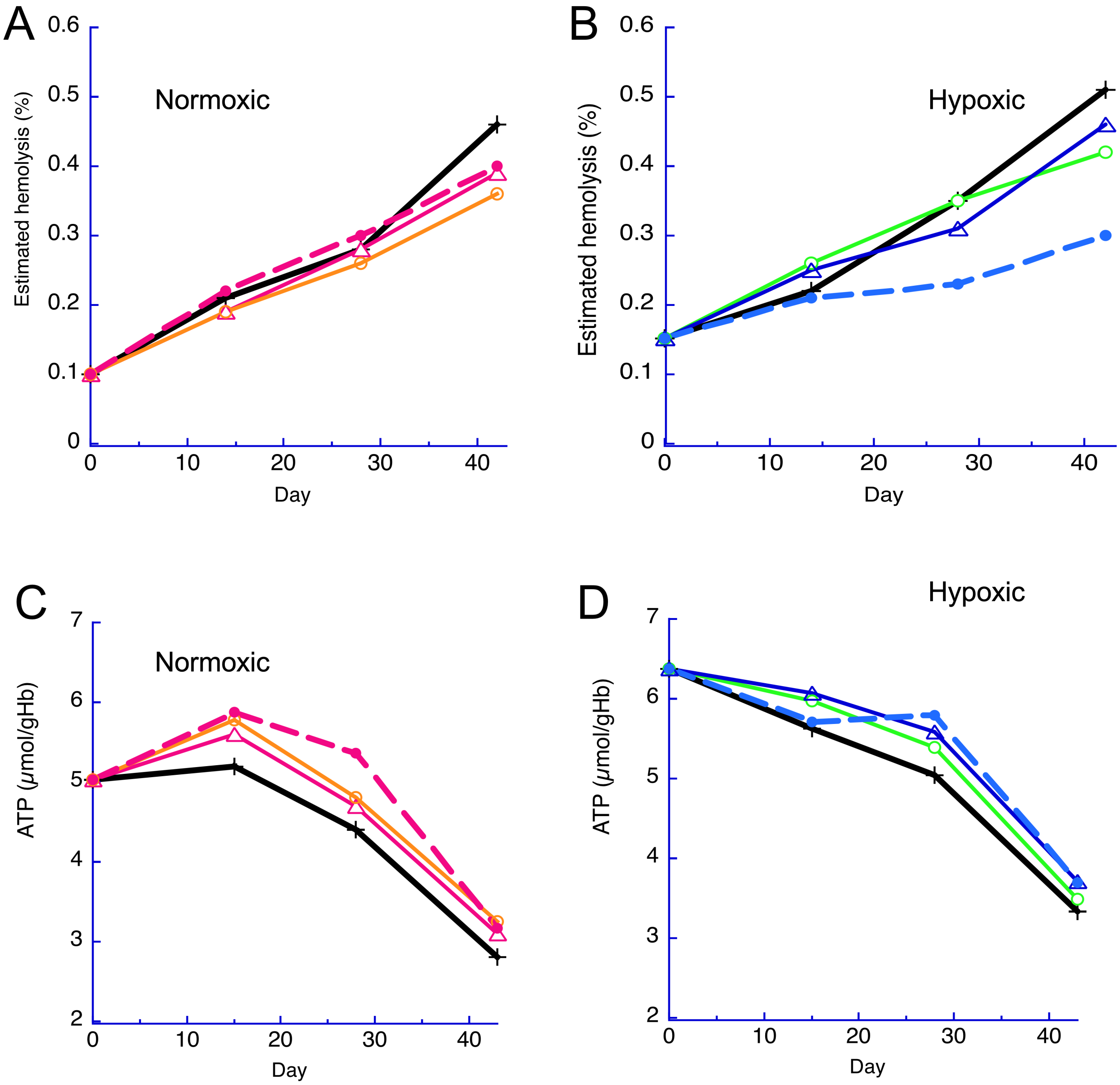

Supplement 1: Estimated hemolysis and ATP: Panel A, estimated hemolysis N RBC; Panel B, estimated hemolysis H RBC; Panel C, ATP N RBC; Panel D, ATP H RBC. Broken lines, PVC bag; open triangle, 2 PVC strips; open circle, 1 PVC strip; cross, no PVC strips.

Supplement 2: Additional details on Methods

PVC strips

The PVC strips were cut from standard PVC blood transfer bags (Fenwal Transfer Pack 4R2032. Fresenius-Kabi, Lake Zurich IL). Approximately 430 cm^2 RBC contact surface was estimated for the standard PVC bag used for storage of LR-RBC (600 mL capacity, Haemonetics, Braintree, MA). Assuming 300 mL of RBC is stored in this bag, 1 mL of RBC contacts 1.4 cm^2 PVC film during storage. Since both sides of the strip is in contact with RBC in the well of the plate, equivalent contact for 2 mL RBC is 1.4 cm^2 of PVC film. Thus, 1 strip of 1cm^2 is equivalent to 71% of PVC exposure.

RBC sample preparation

Sample preparation for the ATP and hemolysis assays was done on day 0, 14, 28 and 42 on ice.

RBC samples were transferred in KDeb Manufacturing (96 Deep Well KDeb Microplate, 2 ml Full Capacity "V" Wells, Polypropylene. Part NO: 850356, Morton MS) and Thermo Fisher 2ml 96 well plates (Nunc™ 96-Well Polypropylene DeepWell™ Storage Plates Cat #: 278752 Thermo Fisher, Waltham MA).

For hemolysis, approximately 1.2 mL of blood was taken from the designated wells in the initial storage plates and replated into the respective wells in the fresh plates for centrifugation. A similar volume was taken from the N and H bags and transferred into remaining wells in the spinning plates. For ATP assay, exactly 0.6 mL of RBC was replated from the initial storage plates into fresh plates. 0.6 mL of 12% of pre-prepared ice-cold trichloroacetic acid (TCA) (T0699-100ml Sigma-Aldrich, MO) was added to the sampled RBCs. After the addition of TCA, the ATP plates were sealed, vigorously shaken for 30 seconds and left on ice for 5 minutes before being placed in the centrifuge. The hemolysis plates were also sealed using the silver adhesive sealer. Both the ATP and hemolysis plates were then centrifuged (Sorvall Legend XFR Centrifuge, Thermo Scientific, Waltham MA) at 3600xG for 10 mins.

After the first spin was completed the supernatant, approximately 0.3 mL, from each of the hemolysis and ATP wells was transferred into their respective wells in another fresh plate with a smaller volume (PP-Masterblock 96 well 0.5 ml plate Greiner Bio-One. REF:786291 LOT:B210133K, NC), resealed and centrifuged again at 3600xG for 10 minutes. After the second spin, exactly 0.2 mL of supernatant from each of the ATP and hemolysis wells was transferred into a fresh plate, sealed, and stored at -80 C for later analysis.

ATP assay

The frozen TCA extracts were thawed on ice at room temperature on the day of analysis. The thawed samples were then assayed using the DiaSys ATP Hexokinase Kit (Cat. No.1 6201 99 10

021, DiaSys Diagnostic Systems GmbH, Germany), ATP Standard FS (Cat. No. 1 6200 99 10 065 DiaSys Diagnostic Systems GmbH, Germany) and the Quantitative In-Vitro Determination of Adenosine-5'-Triphosphate (ATP) assaying procedure adapted to 0.3 ml assay volume for use in a 96-well plate. Spectrophotometric readings were taken with optically clear plates (Costar Assay Plate, 96 Well Polystyrene LOT:05321054 Corning Incorporated, ME) using Biotek.

Estimation of hemolysis

Similarly, the hemolysis samples were thawed at room temperature and supernatant hemoglobin concentration was determined by the Harboe direct spectrophotometric method with Allen correction* in optically clear plates.

Hemolysis was estimated from measured supernatant hemoglobin (sHb) by using a correlation function derived from 411 pairs of sHb vs. spun-hematocrit-based %hemolysis data for leukoreduced RBC stored in AS3, measured at weeks 0, 3, and 6, under both normoxic and hypoxic storage conditions (based on internal study data).

e-hemolysis (estimated) = sHb (mg/dL) * 0.002; (R^2=0.97)

Hemolysis data used for derivation of the correlation function was calculated using spun hematocrit with the standard formula:

% Hemolysis = [sHb (mg/dL) x 0.001) x ((1-(Hct x 0.01)) x 100] / [Total Hb (g/dL)]

Based on the large n, this relationship can be applied specifically for LR-RBC in AS3 for *initial large scale screening purpose.*

*Han V, Serrano K, Devine DV. A comparative study of common techniques used to measure haemolysis in stored red cell concentrates. Vox Sang 2010;98: 116-23.
